# Misalignment between physicians and patient satisfaction with psoriatic arthritis disease control

**DOI:** 10.1007/s10067-017-3578-9

**Published:** 2017-02-25

**Authors:** Daniel E. Furst, Melody Tran, Emma Sullivan, James Pike, James Piercy, Vivian Herrera, Jacqueline B. Palmer

**Affiliations:** 1 Department of Rheumatology, University of California, Los Angeles Medical Center, Los Angeles, CA USA; 2 Scott & White Health Plan, Temple, TX USA; 3 Adelphi Real World, Adelphi Mill, Cheshire, UK; 4 Health Economics & Outcomes Research, Immunology & Dermatology Business Unit, Novartis Pharmaceuticals Corporation, East Hanover, NJ 07936 USA

**Keywords:** Disease activity, Misalignment, Patient-physician survey, Psoriatic arthritis, Swollen joint count, Tender joint count

## Abstract

The main objective of the present study is to evaluate the misalignment between psoriatic arthritis (PsA) patient- and physician-reported satisfaction with PsA control. Data came from the Adelphi Rheumatology Disease Specific Programme, a retrospective, cross-sectional survey of US-based rheumatologists and patients. Physicians provided satisfaction and clinical characteristics on tender joint count, swollen joint count, and percent body surface area (BSA) affected by psoriasis. Patients provided data on satisfaction, the Work Productivity Activity Impairment and Health Assessment Questionnaire-Disability Index (HAQ-DI) questionnaires. Based on their satisfaction response, patient-physician pairs were classified into aligned (both satisfied or dissatisfied) or misaligned (rated satisfaction differently) groups. Multivariate analysis evaluated association of characteristics with misalignment. Among 305 paired patient-physician records analyzed, 23.6% were misaligned and 76.4% were aligned. The misaligned group had shorter disease duration (mean years, 5.2 vs. 6.4), used fewer biologic disease-modifying antirheumatic drugs (49.3 vs. 62.9%), had more swollen (mean, 3.7 vs. 1.9, *P* = 0.0002) and tender joints (mean, 5.6 vs. 2.9, *P* < 0.0001), greater proportion of patients with comorbidities (72.2 vs. 63.1%), and >3% BSA affected by psoriatic skin lesions (64.2 vs. 55.1%). Misaligned patients reported greater work impairment (mean, 38.7 vs. 21.4, *P* = 0.0004), daily activities (mean, 38.7 vs. 22.3, *P* < 0.0001), and higher disease burden (mean HAQ-DI; 0.56 vs. 0.37, *P* = 0.0001). Multivariate analysis found the number of swollen joints (*P* = 0.02) and HAQ-DI score (*P* = 0.03) was significantly associated with misalignment among all patients; however, not in the subgroup of employed patients. Patient-physician misalignment is associated with increased disease activity and disability among patients with PsA.

## Introduction

Psoriatic arthritis (PsA) is a common chronic, disabling, immune-mediated disease, affecting the peripheral and axial joints, nails, and entheses, and is often associated with psoriatic skin lesions [1, 2]. Patients with PsA experience inflammation, pain, and swelling of the joints, in addition to the scaling, itching, and skin pain associated with psoriasis [3]. In the USA, the prevalence of PsA ranges from 0.10 to 0.25%, with approximately 30% of patients with psoriasis developing PsA [2, 4]. Current treatments for PsA focus on reducing inflammation and pain [3, 5, 6]. Treatment of PsA typically involves non-steroid anti-inflammatory drugs, intra-articular corticosteroid injections for mild disease, non-biologic disease-modifying antirheumatic drugs (nbDMARDs), biologics DMARDs (bDMARDs), and a recent classification of targeted synthetic DMARDs (tsDMARDs) [3, 5–10].

The assessment of disease activity in PsA relies partially on patient-reported outcomes in combination with clinical and laboratory evaluation by the physician [11, 12]. Alignment between physicians and patients with respect to PsA activity is important for the optimal implementation of a treatment plan and to promote the most effective outcome for patients [13–17]. The Group for Research and Assessment of Psoriasis and Psoriatic Arthritis suggests that the assessment of PsA activity should include the simultaneous evaluation of arthritis, axial disease, enthesitis, dactylitis, patient and physician global assessment, physical function, health-related quality of life, and skin and nail disease [13–17].

Although validated physician-reported instruments for determining disease activity in PsA have allowed better disease assessment, a number of challenges still exist [18]. PsA symptoms are heterogeneous, and the global disease burden is usually a composite of the different symptoms. In addition, certain symptoms may have a greater influence on the perception of PsA activity, which may differ between the patient and physician [18]. A patient’s point of view is typically based on their experience with PsA over a long period of time, while a physician’s perception of PsA activity is related to his or her professional experience [18].

A limited number of studies have evaluated misalignment between patients and physicians with regard to PsA activity. Findings from these studies suggest a significant disconnection in the manner in which PsA patients and their rheumatologists define and report PsA activity and control [11, 18, 19]. Currently available data also indicate low patient satisfaction with care among some patients with PsA and has been associated with a lack of psychological support and knowledge about PsA and treatment [20]. Hence, the aim of this study was to ascertain the extent of misalignment between patient- and physician-reported satisfaction with PsA control and its association with PsA activity and disease burden. In patients who had active joint disease, an exploratory analysis was undertaken to describe and compare the characteristics of patients who were satisfied or dissatisfied with their current PsA control.

## Methods

### Data source

This analysis used data retrieved from the Adelphi Disease Specific Programme, a large, syndicated, retrospective, multinational surveys of physicians and patients in a real-world clinical setting for a range of common diseases [21]. The Disease Specific Programme collects quantitative and qualitative survey data and provides a comprehensive overview of a given disease and treatment of that disease from the perspective of both physician and patient [21]. Two Rheumatology Disease Specific Programme surveys conducted in the USA between January and March 2011 and over a similar time period in 2014, were used for this study. The Disease Specific Programme included a geographically diverse sample of US rheumatologists and their respective patients with PsA. The Rheumatology Disease Specific Programme was conducted in accordance with the US Health Insurance Portability and Accountability Act 1996 (HIPAA; www.hhs.gov/ocr/privacy/) and the Health Information Technology for Economic and Clinical Health legislation (2014 only as this legislation was not present in 2011). The Disease Specific Programme is a market research project and complies with all relevant market research guidelines and legal obligations. The research methodology and nature of the collected data make submission to national and/or local ethics committees and regulatory bodies unnecessary. Namely, the Disease Specific Programme is non-interventional and employs solely a retrospective data collection, and both physician and patient data are collected anonymously and independently.

Physicians were identified from public lists of healthcare professions. The physician sample included 200 US rheumatologists (100 sampled in each year) responsible for managing patients with PsA. Eligible physicians had to meet the following pre-specified criteria: primary specialty was rheumatology, currently treating rheumatoid arthritis (RA), PsA, and spondyloarthropathy; typical monthly workload involved consultations with three or more patients with PsA; and qualification as a physician between 3 and 40 years prior to initiation of the survey.

Each physician completed a response form for three consecutive, consulting, adult patients with PsA, generating 600 forms across the two surveys. Eligible patients had to be ≥18 years of age and have a diagnosis of PsA on or before the day of consultation. Patients were excluded if they were involved in a clinical trial. All patients gave their informed consent. Subjects had the right to opt-out of the survey at any time.

### Survey design

The Rheumatology Disease Specific Programme was developed by Adelphi Real World (Adelphi Real World, Cheshire, UK). All physician-completed patient record form answers were confidential and maintained physician and patient anonymity; data were fully de-identified prior to receipt by the research team. The physician-completed patient record form provided information on a wide a range of patient and disease characteristics, including demographics, comorbidities, symptomatology, and satisfaction with PsA control. All data from the physician-completed patient record form were based on evidence available to the physician at the time of the consultation; no tests or investigations were performed as part of this research.

Patients were asked to fill out patient self-completed questionnaires on a voluntary basis. To preserve anonymity, patients were asked to complete the form independently of the physician and return the patient self-completed questionnaires in a sealed envelope. To ensure the physician did not see any patient responses (including patient-reported outcome measures and symptom assessments), the patient put their responses in an envelope and sealed this prior to return. Each pair of forms (i.e., the physician-completed patient record form and the patient self-completed questionnaire) was linked during data processing using non-identifying unique identification numbers. All eligible pairs of linked physician-completed patient report forms and the patient self-completed questionnaires were included for analysis.

**Fig. 1:**
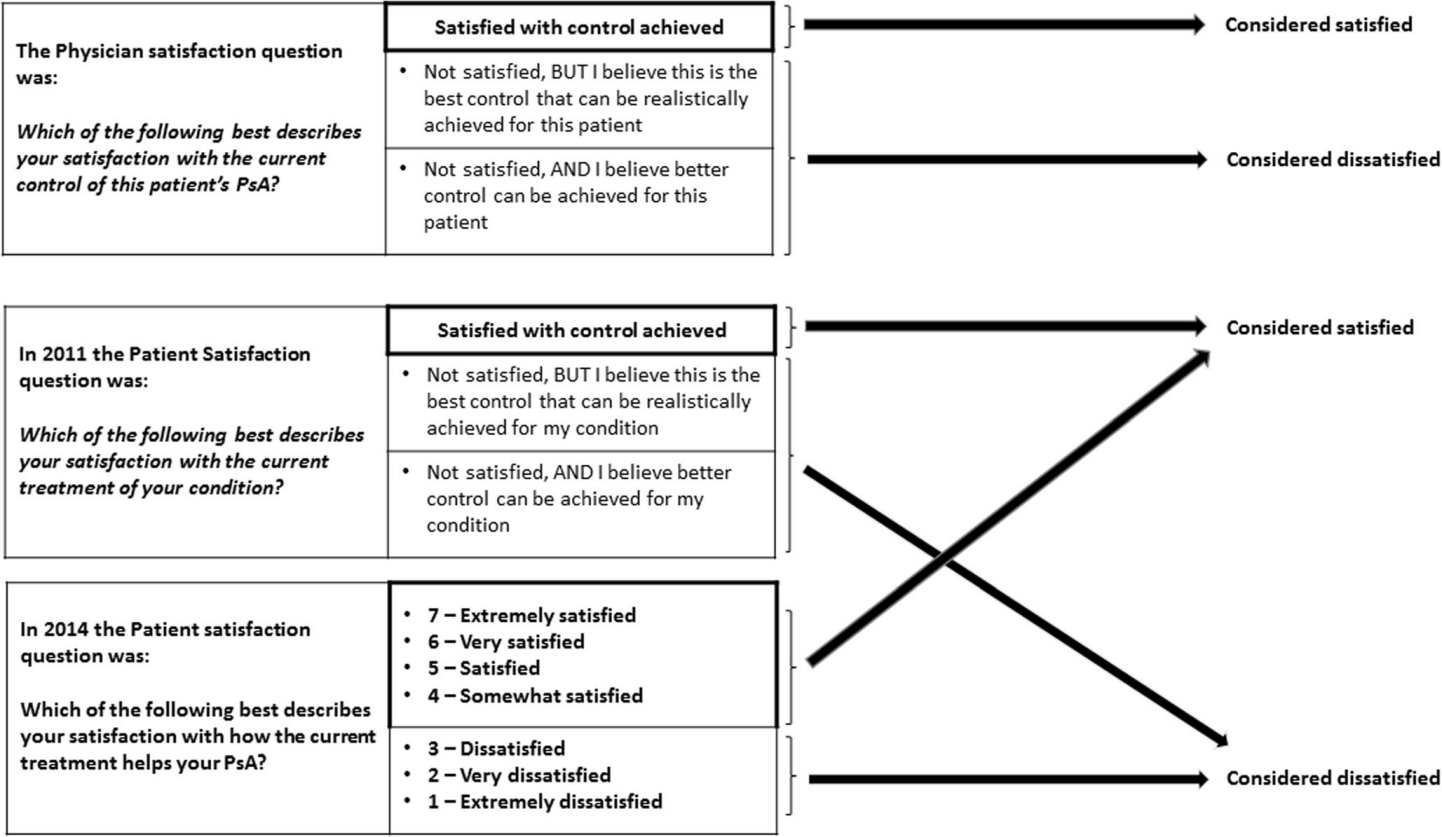
Determination of whether physicians and patients were satisfied or dissatisfied with control. (*Note*: Physicians and patients responded to specific questions, and depending upon their response were considered satisfied or dissatisfied with PsA control; *PsA* psoriatic arthritis)

Patients reported their satisfaction with PsA control; in 2011, this was in response to a categorical question, and in 2014, this was captured as a response to a Likert scale (Fig. 1).

The validated Work Productivity Activity Impairment (WPAI) [20] and Health Assessment Questionnaire-Disability Index (HAQ-DI) (HAQ-DI max = 3.0) [21] questionnaires were included in the patient self-completed questionnaires for completion by patients, allowing scores to be derived for both measures. The overall percentage of “work impairment” as well as the percentage of “presenteeism” and “absenteeism” was calculated for patients who were employed at the time of the survey. However, the percentage of “activity impairment” derived from the WPAI responses was calculated for the whole sample (both employed and unemployed patients).

#### Determination of misalignment on satisfaction

The responses to the satisfaction questions from each pair of linked forms (physician-completed patient record form and patient self-completed questionnaire) were compared to determine if the physician and patient pair were ‘aligned’ or ‘misaligned’ in terms of their satisfaction with PsA control. Pairs were classified as ‘aligned’ when both the patient and physician felt satisfaction or dissatisfaction in terms of PsA control or as ‘misaligned’ when the physician felt satisfied, but the patient was dissatisfied with PsA control or vice versa.

### Variables

Study variables of interest included physician and patient reported satisfaction with PsA control, demographics characteristics, disease characteristics, and burden of disease. The following variables were captured from physicians in the physician-completed patient record form: satisfaction with PsA control (categorized as satisfied or dissatisfied), patient age, sex, comorbidities (listed below), time since diagnosis measured in years, current treatment (e.g., topical agents, nbDMARDs, bDMARDs), tender joint count (TJC), swollen joint count (SJC), percent body surface area (BSA) affected by psoriatic skin lesions, and number of PsA symptoms currently present including joint symptoms (tenderness, swelling, stiffness, etc.) and skin symptoms for those with psoriatic skin lesions (itching, pain, scaling, etc.). Comorbidities included anxiety, depression, type 2 diabetes, hyperlipidemia/elevated cholesterol, gastric condition, hypertension, liver impairment, malignancy, obesity, renal impairment, osteoporosis, respiratory conditions, tuberculosis, and vasculitis. The patient self-completed questionnaire captured satisfaction with PsA control (categorized as satisfied or dissatisfied), WPAI (a percentage of overall work impairment scored as 0 to 100% impairment and impairment of “presenteeism”, “absenteeism”, and activity impairment) [20], and HAQ-DI (continuous variables of scores ranging from 0 to 3 with a score of 0 indicating performance without any difficulty and up to score 3 meaning performance cannot be done at all) [21].

### Data analysis

Data were reported descriptively for each variable (i.e., patient age, sex, comorbidities, time since diagnosis, current treatment, TJC, SJC, percent BSA affected by psoriatic skin lesions, number of PsA symptoms currently present, HAQ-DI scores, and WPAI responses). Categorical variables were summarized using frequency counts and percentages. Continuous variables were summarized by the number of observations, their mean, and standard deviation (SD).

Bivariate statistical comparisons were made between the aligned and misaligned groups for each variable. *P* values were obtained using the Wilcoxon rank-sum test for continuous variables, and Fisher’s exact test for categorical variables.

For the primary objective, multivariate logistic regression analyses evaluated what factors may be associated with patient and physician misalignment. Two analyses were performed as follows: the first included all patient-physician pairs and the second included the subgroup of patients who were employed and had completed the WPAI. The dependent variable was whether patients were aligned with their physicians in regard to satisfaction of PsA control. Independent variables included age, current bDMARD treatment, SJC, percent BSA affected by psoriatic skin lesions, and HAQ-DI. For the multivariate analysis, TJC was omitted from the model because there was multicollinearity with SJC. In addition, TJC is confounded by other diseases, such as osteoarthritis, and is an indirect measure of inflammation [22]. SJC, on the other hand, is a good measure of inflammation [23]. All variables were included in the models at the same time. Standard errors were adjusted to allow for possible intragroup correlation within the reporting physician. The multivariate logistic regression was repeated on the subpopulation of patients who had completed the WPAI using WPAI as an independent variable to identify independent predictors of misalignment of employed patients.

### Sub-analysis of satisfaction with PsA control in patients with active joint disease

An exploratory analysis was performed in two groups of patients with active joint disease (>3 TJC): satisfied and not satisfied with PsA control. Data were reported descriptively for each variable. Categorical variables were summarized using frequency counts and percentages. Continuous variables were summarized by the number of observations, the mean, and SD.

Bivariate statistical comparisons were made between the satisfied and not satisfied groups for each variable. *P* values were obtained using the Wilcoxon rank-sum test for continuous variables, and Fisher’s exact test for categorical variables.

## Results

### Survey physician and patient population

A total of 327 patients completed a patient self-completed questionnaire and were included in the analysis. Patients were matched with their respective rheumatologist, who completed a physician-completed patient record form. Twenty-two records were excluded due to missing data relating to satisfaction from either the patient or the physician. Therefore, 305 paired rheumatologists and PsA patient records were eligible for inclusion in the analysis.

Of the complete set of patient-physician records, 76.4% were “aligned”, with 65.2% in the aligned group being both satisfied and 11.1% being both dissatisfied with PsA control (Table 1). The remaining 23.6% of patient-physician records were “misaligned”. In the misaligned group, 17.0% of the paired patient-physician records consisted of a satisfied patient and dissatisfied physician, and 6.6% consisted of a dissatisfied patient and satisfied physician.

**Table 1:** Paired patient-physician survey responses

Aligned patient-physician records, pairs, *n* (%)	233 (76.4)
Patient and physician both satisfied	199 (65.2)
Patient and physician both dissatisfied	34 (11.1)
Misaligned patient-physician records, pairs, *n* (%)	72 (23.6)

### Baseline demographics and disease characteristics

The misaligned group had greater disease activity compared with that of the aligned group. The aligned and misaligned groups were similar with regard to age and gender (Table 2). In the misaligned group, patients had a shorter disease duration (mean years [SD] 5.2 [5.3] vs. 6.4 [7.1]) and a greater percentage were not using bDMARD therapy (50.7 vs. 37.1%) compared with those of the aligned group. Patients in the misaligned group also tended to have more active disease than those of the aligned group, with a significantly higher number of swollen (mean [SD], 3.7 [4.0] vs. 1.9 [3.1], *P* = 0.0002) and tender joints (mean [SD], 5.6 [5.5] vs. 2.9 [3.7], *P* < 0.0001) and a greater percentage of patients had >3% of their BSA affected by psoriatic skin lesions (64.2 vs. 55.1%). The misaligned group also had a significantly greater number of PsA symptoms present (mean [SD], 6.8 [3.8] vs. 4.9 [3.6], *P* = 0.0004) as well as a larger percentage of patients with >5 symptoms (65.3 vs. 40.8%, *P* = 0.0004) compared with those of the aligned group. The most common comorbidities across both groups were hypertension (28.9%), elevated cholesterol (20.0%), depression (14.1%), obesity (13.8%), and anxiety (10.8%). Compared with that of the aligned group, a greater percentage of patients in the misaligned group had comorbidities (72.2 vs. 63.1%), including depression (20.8 vs. 12.0%) and anxiety (15.3 vs. 9.4%) (Table 2).

In regard to work productivity, the misaligned group was significantly more impaired by PsA in their overall work (mean % [SD], 38.7 [27.9] vs. 21.4 [26.7], *P* = 0.0004), while at work (mean % [SD], 36.2 [25.3] vs. 16.5 [21.2], *P* < 0.0001) and in their daily activities (mean % [SD], 38.7 [24.5] vs. 22.3 [24.0], *P* < 0.0001) compared with that of the aligned group. The misaligned group had a significantly higher disease burden as measured by HAQ-DI (mean [SD], 0.56 [0.43]) than that of the aligned group (mean [SD], 0.37 [0.48]) (*P* = 0.0001) (Table 2).

**Table 2:** Baseline demographics and disease characteristics

	Overall, *N* = 305	Aligned, *n* = 233	Misaligned, *n* = 72	*P* value^a^
Age (*y*), mean (SD)	50.0 (13.4)	50.0 (13.5)	49.8 (13.1)	0.99
Male, *n* (%)	168 (55.1)	129 (55.4)	39 (54.2)	0.89
Time since diagnosis (*y*), mean (SD)	6.1 (6.7)	6.4 (7.1)	5.2 (5.3)	0.28
Current bDMARD treatment, *n* (%)				
None	122 (40.3)	86 (37.1)	36 (50.7)	0.05
Currently receiving bDMARD treatment	181 (59.7)	146 (62.9)	35 (49.3)	
SJC, mean (SD)	2.4 (3.4)	1.9 (3.1)	3.7 (4.0)	0.0002
TJC, mean (SD)	3.5 (4.4)	2.9 (3.7)	5.6 (5.5)	<0.0001
BSA affected, *n* (%)				
≤3%	121 (42.8)	97 (44.9)	24 (35.8)	0.21
>3%	162 (57.2)	119 (55.1)	43 (64.2)	
Number of PsA symptoms, mean (SD)^b^	5.4 (3.8)	4.9 (3.6)	6.8 (3.8)	0.0004
Number of PsA symptoms, *n* (%)^b^				
≤5	163 (53.4)	138 (59.2)	25 (34.7)	0.0004
>5	142 (46.6)	95 (40.8)	47 (65.3)	
Comorbidities				
Number of comorbidities per patient, mean (SD)	1.2 (1.3)	1.1 (1.3)	1.4 (1.4)	0.11
Frequency of comorbidities (≥1), *n* (%)	199 (65.2)	147 (63.1)	52 (72.2)	0.20
Comorbidities, *n* (%)				
Hypertension	88 (28.9)	67 (28.8)	21 (29.2)	>0.9999
Hyperlipidemia/elevated cholesterol	61 (20.0)	49 (21.0)	12 (16.7)	0.50
Depression	43 (14.1)	28 (12.0)	15 (20.8)	0.08
Obesity	42 (13.8)	30 (12.9)	12 (16.7)	0.44
Anxiety	33 (10.8)	22 (9.4)	11 (15.3)	0.19
Gastric condition	31 (10.2)	22 (9.4)	9 (12.5)	0.50
Type 2 diabetes	28 (9.2)	18 (7.7)	10 (13.9)	0.16
Liver impairment	8 (2.6)	5 (2.1)	3 (4.2)	0.40
Malignancy	7 (2.3)	6 (2.6)	1 (1.4)	>0.9999
Respiratory condition	7 (2.3)	5 (2.1)	2 (2.8)	0.67
Osteoporosis	6 (2.0)	4 (1.7)	2 (2.8)	0.63
Renal impairment	5 (1.6)	4 (1.7)	1 (1.4)	>0.9999
Tuberculosis	2 (0.7)	2 (0.9)	0 (0)	>0.9999
WPAI due to PsA, mean (SD)				
Percentage of work-time missed	6.3 (17.3)	6.3 (17.0)	6.4 (18.3)	0.91
Percentage of impairment while working	20.7 (23.4)	16.5 (21.2)	36.2 (25.3)	<0.0001
Overall percentage of work impairment	25.6 (27.9)	21.4 (26.7)	38.7 (27.9)	0.0004
Percentage of activity impairment	26.0 (25.0)	22.3 (24.0)	38.7 (24.5)	<0.0001
HAQ-DI, mean (SD)	0.42 (0.48)	0.37 (0.48)	0.56 (0.43)	0.0001

*bDMARD* biologic disease-modifying antirheumatic drug, *BSA* body surface area, *HAQ*-*DI* health assessment questionnaire disability index, *PsA* psoriatic arthritis, *S* standard deviation, *SJC* swollen joint count, *TJC* tender joint count, *WPAI* work productivity and activity impairment, *y* years

^a^ *P* values were obtained using the Wilcoxon rank-sum test for numeric variables, and Fisher’s exact test for categorical variables

^b^Number of PsA symptoms currently present including joint symptoms tenderness, swelling, stiffness, etc.

### Multivariate analysis: patient-physician misalignment

#### Satisfaction with PsA control in overall population

Multivariate analysis was performed to assess what characteristics were associated with misalignment. After controlling for baseline characteristics, the SJC and the HAQ-DI scores were significantly associated with misalignment (Table 3, Model 1). Multivariate analysis reported that with a higher SJC or HAQ-DI score, the likelihood of being misaligned also increased. When including only employed patients who completed the WPAI in the multivariate analysis, no variables were found to be significantly associated with misalignment (Table 3, Model 2).

**Table 3:** Factors associated with misalignment and subgroup analysis of employed patients with WPAI results^a^

	Model 1^b^		Model 2 (WPAI)	
Variable	OR (95% CI)	*P* value	OR (95% CI)	*P* value
Age	1.00 (0.97–1.03)	0.857	1.00 (0.95–1.07)	0.753
Current bDMARD	0.83 (0.40–1.73)	0.621	0.54 (0.15–1.97)	0.347
SJC	1.13 (1.02–1.26)	0.020	1.16 (0.97–1.40)	0.103
BSA > 3%	0.62 (0.30–1.29)	0.205	0.81 (0.27–2.41)	0.701
HAQ-DI	2.51 (1.12–5.61)	0.025	3.44 (0.88–13.39)	0.074
WPAI			1.01 (0.99–1.03)	0.369

*bDMARD* biologic disease-modifying antirheumatic drug, *BSA* body surface area, *CI* confidence interval; *HAQ*-*DI* health assessment questionnaire disability index, *OR* odds ratio, *SJC* swollen joint count, *TJC* tender joint count, *WPAI* work productivity and activity impairment

^a^Number of observations was 196 and 92 for models 1 and 2, respectively. Continuous variables were patient age (18 to 89 years), SJC (0 to 28), HAQ-DI (0 to 3), and WPAI (0 to 100). Categorical variables were current bDMARD treatment (bDMARD/no bDMARD) and BSA (>3/≤3)

^b^TJC was excluded from the model due to the issue of multicollinearity with SJC

#### Satisfaction with PsA control in patients with active disease (>3 tender joint count)

To assess factors associated with satisfaction in PsA in patients who have active joint disease, we performed an exploratory comparison of the characteristics of patients with active joint disease (>3 TJC) who were satisfied or dissatisfied with PsA control. A subset of 78 patients of the total population was identified from the database currently with active joint disease. Overall, the majority of patients with active disease were satisfied with their PsA control (Table 4). Compared with dissatisfied patients with active joint disease, satisfied patients tended to be older (mean [SD], 53.5 [12.3] vs. 44.3 [10.0] years of age, *P* = 0.001, respectively), male (59.3% vs. 50.0%) had a longer time since PsA diagnosis (mean year [SD], 6.5 [7.6] vs. 3.6 [3.9]), were more likely to be receiving bDMARD therapy (64.8 vs. 56.5%), and had anxiety (13 vs. 33.3%, *P* = 0.06) (Table 4). The level of PsA disease activity as measured by SJC and TJC was similar between satisfied and unsatisfied patients, although percentage of patients with >3% BSA affected by psoriatic lesions was lower in the satisfied patient group (71.4 vs. 82.6%). The HAQ-DI score was also similar between groups (mean [SD], 0.66 [0.50] vs. 0.76 [0.60]) (Table 4).

**Table 4:** Baseline demographics and disease characteristics of PsA patients with active disease (>3 TJC)

Variable	Overall, *N* = 78	Not satisfied, *n* = 24	Satisfied, *n* = 54	*P* value^b^
Age (*y*), mean (SD)	50.6 (12.4)	44.3 (10.0)	53.5 (12.3)	0.001
Male, *n* (%)	44 (56.4)	12 (50.0)	32 (59.3)	0.469
Time since diagnosis (*y*), mean (SD)	5.6 (6.9)	3.6 (3.9)	6.5 (7.6)	0.174
Current bDMARD treatment, *n* (%)				0.609
None	29 (37.7)	10 (43.5)	19 (35.2)	
Receiving bDMARD treatment	48 (62.3)	13 (56.5)	35 (64.8)	
SJC, mean (SD)	5.1 (4.1)	5.0 (3.4)	5.1 (4.4)	0.681
TJC, mean (SD)	7.9 (4.5)	7.8 (3.4)	7.9 (4.9)	0.628
BSA affected, *n* (%)				0.340
≤3%	18 (25.0)	4 (17.4)	14 (28.6)	
>3%	54 (75.0)	19 (82.6)	35 (71.4)	
Number of PsA symptoms, mean (SD)	7.4 (3.7)	8.2 (3.4)	7.1 (3.8)	0.130
Number of PsA symptoms, *n* (%)				0.195
≤5	25 (32.1)	5 (20.8)	20 (37.0)	
>5	53 (67.9)	19 (79.2)	34 (63.0)	
Comorbidities				
Number of comorbidities per patient, mean (SD)	1.8 (1.6)	2.2 (1.8)	1.6 (1.5)	0.130
Frequency of comorbidities (≥1), *n* (%)	61 (78.2)	20 (83.3)	41 (75.9)	0.562
Comorbidities, *n* (%)				
Hypertension	24 (30.8)	9 (37.5)	15 (27.8)	0.432
Obesity	20 (25.6)	5 (20.8)	15 (27.8)	0.586
Depression	18 (23.1)	8 (33.3)	10 (18.5)	0.243
Hyperlipidemia	17 (21.8)	7 (29.2)	10 (18.5)	0.374
Type 2 diabetes	16 (20.5)	3 (12.5)	13 (24.1)	0.364
Anxiety	15 (19.2)	8 (33.3)	7 (13.0)	0.06
Gastric condition	14 (17.9)	6 (25.0)	8 (14.8)	0.342
Respiratory condition	5 (6.4)	3 (12.5)	2 (3.7)	0.166
Malignancy	4 (5.1)	1 (4.2)	3 (5.6)	>0.999
Renal impairment	3 (3.8)	2 (8.3)	1 (1.9)	0.223
Liver impairment	2 (2.6)	1 (4.2)	1 (1.9)	0.524
WPAI due to PsA, mean (SD)^a^				
Percentage of work-time missed	12.6 (21.0)	8.1 (9.1)	14.0 (23.5)	0.738
Percentage of impairment while working	32.7 (24.0)	32.9 (23.3)	32.6 (24.6)	0.921
Overall percentage of work impairment	39.9 (28.1)	40.5 (23.8)	39.7 (29.6)	0.710
Percentage of activity impairment	41.4 (22.1)	47.9 (21.3)	38.5 (22.0)	0.114
HAQ-DI, mean (SD)	0.69 (0.53)	0.76 (0.60)	0.66 (0.50)	0.410

*bDMARD* biologic disease-modifying antirheumatic drugs, *BSA* body surface area, *HAQ*-*DI* health assessment questionnaire disability index, *PsA* psoriatic arthritis, *SD* standard deviation, *SJC* swollen joint count, *TJC* tender joint count, *WPAI* work productivity and activity impairment; *y* years

^a^WPAI was not available for all patients evaluated. Thirty-eight employed patients provided overall WPAI scores; nine from the not satisfied group and 29 from the satisfied group

^b^P values were obtained using the Wilcoxon rank-sum test for numeric variables, and Fisher’s exact test for categorical variables

## Discussion

Unlike prior studies, we surveyed a geographically diverse sample of patients and physicians in a real-world clinical setting in the USA. This analysis is also distinctive because it is one of the first studies to assess the relationship between patient-physician misalignment and PsA disease activity, describing detailed demographic and disease patient characteristics in relation to the examined types of patients.

In this research, approximately 25% of physicians and their PsA patients were misaligned with regard to their satisfaction of PsA control, with the majority of misaligned cases consisting of a satisfied patient and dissatisfied physician. The misaligned group reported significantly more PsA symptoms, indicating increased PsA activity compared with that of the aligned group. The percentage of patients with comorbidities was also higher in the misaligned than that of the aligned group. While some indicators of disease activity were not significant, the number of differences and their consistency leads the authors to believe that the misaligned group had slightly more severe and active disease and were generally more complex in presenting with more comorbidities. In addition, the majority of misalignment was due to physician dissatisfaction with their patient’s PsA control. This misalignment may be an indicator that the treatment goals may or may not have been aligned between physicians and patients (i.e., reduced disease activity vs. remission).

Multivariate analysis found that a greater SJC and a higher HAQ-DI score were significantly associated with patient-physician misalignment. However, in the patient-physician group in which patients were employed and had completed the WPAI, there were no variables significantly associated with misalignment. The lack of significance may be due to the smaller number of employed patients and within this decreased sample size, only 38 patients had available WPAI information. The findings from this exploratory analysis suggest that misalignment may be associated with more active disease and poorer PsA control, increased comorbidities, more extensive disability, and unemployment. Nevertheless, the majority of patients with active disease were satisfied with their PsA control.

Our findings are consistent with prior smaller single-center studies that assessed factors that may influence the differences in patient-physician alignment with regard to PsA activity and control [11, 18, 19]. Previous studies found that patients with PsA experience a more severe burden of disease than that perceived by the physician [11, 18], and nearly one quarter to a third of patients with PsA were misaligned with their physicians [11]. The earlier studies found greater misalignment regarding the perception of disease activity in the joints than that of the skin symptoms (i.e., TJC, SJC vs. psoriasis, etc.) [11, 18, 19], possibly indicating that skin lesions are more obvious and easily perceived, so are easier to “align” [19]. In previous PsA studies, factors associated with misalignment were SJC, TJC, pain, and fatigue [11, 18, 19]. Increased TJC and SJC resulted in worse physician assessment of arthritis [11]. A meta-analysis found that the number of swollen and tender joints influenced the perception of disease activity. Patient-reported and trained observer assessment for SJC showed lower levels of correlation than those of patient-reported TJC [24].

In one study, pain and fatigue were the two major causes for misalignment and resulted in worse patient assessment of their disease [11]. We did not assess the association between pain and fatigue with patient and physician misalignment in this study. In previous PsA studies, misalignment was predominately in patients with worse self-rating of overall disease activity [11, 18]. Differences in ratings of disease activity between physicians and patients may be influenced by a number of factors such as lower education level, being a smoker, being unemployed, and experiencing depression, anxiety, and fibromyalgia [11]. This study analyzed some of these associations in a bivariate manner; however, we did not analyze these associations with misalignment in the multivariate model due to the relatively small number of misaligned completed surveys and validity concerns.

Satisfaction is seen as an indicator of quality of health care [25, 26]. In our study, about 69% of patient with active disease were still satisfied with control of their PsA. Though speculative in nature, these findings suggest that patients with active disease may “settle” for suboptimal control of joint activity based on their previous experience, particularly in patients with longer disease duration and bDMARD use [27]. A recent study reported higher treatment satisfaction among patients than that of physicians and noted that patients may assess disease severity differently from physicians by considering symptoms that may not be captured during a physician visit [28]. Other factors found to influence satisfaction with care in patients with PsA include involvement in healthcare decision-making, and access to health care services, particularly rheumatology services, and adequacy of health care facilities [20, 29, 30]. These factors were not available for analysis in our data.

The challenges in evaluating disease activity have been described in numerous survey-based RA studies, where misalignment between patient and physician global disease assessment was reported in approximately 30% of patients with RA [31–34]. Alignment between patient and physician satisfaction of disease control improves the chances of a treatment plan being successfully implemented [13]. A patient-centered approach in managing chronic illnesses, where patients participate and share activity in treatment and management of the disease that takes into account individual preference with social context [4], helps to promote the alignment of the patient and physician on treatment and disease management [13]. Patients involved in decision-making appear to have better outcomes and are more likely to be satisfied with their health care [29, 30]. The treat-to-target approach asks the practitioner to treat towards the goal of remission or minimal disease activity [14, 16]. The current study finds that, in some cases, even though some patients had some disease activity (i.e., increased swollen joint counts), they were still satisfied. In other instances, patients were satisfied with their PsA control, whereas the physician was not. This idea of misalignment in disease control may add important information for decisions made by physicians using the currently recommended treat-to-target approach for managing PsA [16]. It also encourages both the patient and physician to align on a similar treatment goal.

There are several limitations to this analysis. The sample collected in the Disease Specific Programme is not a truly random sample of patients. Patients included in the Disease Specific Programme sample were the next three patients with PsA who consulted the physician. While a reasonable approach, it may not truly represent the overall population of patients with PsA, as patients who consult frequently are more likely to be included in the sample. However, the patients are representative of the patient population who consult rheumatologists. This study used the 28 TJC which may have been less specific for PsA than the 66 SJC and the 68 TJC. We only included SJC and did not include TJC in our multivariate analysis, specifically because we wished to include patients most likely to have true inflammatory disease. The methodology used in the Disease Specific Programme has some limitations that are common to all survey-based methodologies including potential recall bias, possible physician selection bias (the survey focused on rheumatologists who saw at least a minimum number of PsA patients), physician willingness to fill out a survey themselves, and potential biases engendered by the specific questionnaires used which may not reflect all aspects of individual impairment from the patient’s point of view [18]. In common with any research where participation is voluntary including clinical trials, inclusion of patients and physicians may also be subject to bias.

Future research should include a large sample of PsA patients and paired-provided survey responses to further investigate the reasons for dissatisfaction and satisfaction among patients and providers. Among the misaligned patients and providers, a comparison should be conducted to separately examine the satisfied patients and dissatisfied providers and vice versa.

## Conclusion

In conclusion, our findings in a diverse PsA population indicate that about a quarter of patients with PsA are misaligned with their rheumatologists in their satisfaction with their PsA control. Patient-physician misalignment is associated with increased disease activity and disability among patients with PsA. Our findings stress the importance of strong and effective communication between patients and their physicians in treating this chronic disease.
